# Hypertension Control Rate, Associated Factors, Facilitators, and Barriers in Kerala, India: A Mixed-Methods Study

**DOI:** 10.7759/cureus.91073

**Published:** 2025-08-26

**Authors:** Das P Anaswara, Sunu C Thomas, Jeby J Olickal, P Sankara Sarma, Kavumpurathu R Thankappan

**Affiliations:** 1 Department of Public Health, Amrita Institute of Medical Sciences, Amrita Vishwa Vidyapeetham, Kochi, IND

**Keywords:** associated factors, barriers, facilitators, hypertension control, kerala

## Abstract

Introduction

Comprehensive studies on the hypertension control rate are limited in India. We assessed the hypertension control rate, associated factors, facilitators, and barriers in Kerala, India.

Methods

We surveyed 256 hypertensive adults aged ≥30 years (males, 45%) selected using multistage cluster sampling. Log-binomial regression was done to find the factors associated with hypertension control. We conducted in-depth interviews with 11 hypertensives and 14 healthcare providers.

Results

Hypertension control rate was 23.04% (95% confidence interval (CI): 18.31-28.58%). Better control was seen in those aged ≥60, females, and those reporting healthy diets, physical activity, no abdominal obesity, and no comorbidities. Key facilitators included community programs, affordable care, awareness, and family support, while barriers included poor access, medicine shortages, distrust, misconceptions, and lack of family support.

Conclusion

Improving antihypertensive access, addressing misconceptions, and promoting diet and physical activity, with a focus on males and younger adults, are likely to enhance hypertension control in Kerala.

## Introduction

In 2019, 1.27 billion people aged 30-79 years had hypertension globally, with a prevalence of 32% in women and 34% in men [1]. The number is projected to reach 1.56 billion by 2025 [2]. High-income countries saw a slight decline, whereas low- and middle-income countries (LMICs) experienced a significant increase over two decades [3]. Detection (women, 59%,;men, 49%), treatment (women, 47%; men, 38%), and control (women, 23%; men, 18%) rates were higher in women [1]. In 2021, hypertension was the leading risk factor for premature mortality [4], causing 8.5 million deaths in 2019 from stroke, ischemic heart disease, renal disease, and other vascular conditions [1]. High systolic BP was the primary contributor to cardiovascular disease (CVD) disability adjusted life years (DALYs), with 2,564.9 per 100,000 persons in 2022. The proportion of chronic heart disease (CHD) and stroke attributed to high blood pressure (BP) was 49% and 62%, respectively [1]. In India, 35.5% of adults (315 million) have hypertension [5], with awareness, treatment, and control rates of 27.9%, 14.5%, and 12.6%, respectively [6]. Cases increased from 118 million in 2000 [7] to 315 million in 2020 [5].

Prevalence varied across states, from 24.3% in Meghalaya to 51.8% in Punjab [5], with significant disparities at state, district, and urban-rural levels [8]. Significant disparities existed among districts in detecting (6.3%-77.5%), treating (8.7%-97.1%), and controlling blood pressure (2.7%-76.6%) [2].

Kerala is the most advanced Indian state in epidemiological transition [9] with a hypertension prevalence of 47.6%, second only to Punjab (51.8%) [5]. Among adults aged 20-79 years, awareness, treatment, and control rates were 44.7%, 35.8%, and 18.3% respectively [10]. Control rates were 24% [11] for those aged ≥60 years and 12.5% [10] for 20-39 years old. Despite Kerala’s superior health indicators, infant mortality (six per 1,000 live births vs. India’s 28) and life expectancy (75 years, comparable to high-income countries), its hypertension control rate (12.4%) remains low [10]. Kerala shows wide district-level variation in hypertension control, with significant heterogeneity in awareness, treatment, and outcomes. Selecting two districts with contrasting profiles is sufficient to capture this variability and generate insights applicable across similar settings within the state and other regions undergoing similar health transitions.

A qualitative study in Pathanamthitta District, Kerala, identified several patient-level factors for poor hypertension control: inadequate understanding, self-medication, healthcare inaccessibility, lack of social support, high costs, distrust in public healthcare, unfriendly provider attitudes, irregular medicine supply, and pill burden [12]. Provider perspectives remain underexplored, and factors associated with hypertension control in Kerala’s adult population are not well studied. Therefore, this study aimed to assess the hypertension control rate, associated factors, facilitators, and barriers in Kerala.

## Materials and methods

Study design

A mixed-methods approach with a sequential explanatory design was used to understand the factors associated with hypertension control. The study comprised two phases: an initial quantitative phase involving a community-based cross-sectional survey among adults aged 30 years and above, followed by a qualitative phase consisting of in-depth interviews with individuals with uncontrolled hypertension identified in the quantitative phase, as well as frontline healthcare providers.

Study setting and participants

The study was conducted in two Kerala districts: Kannur (northern region) and Ernakulam (southern region). The quantitative phase included adults aged ≥30 years with hypertension (systolic blood pressure (SBP) ≥140 mmHg and/or diastolic blood pressure (DBP) ≥90 mmHg or on medication for hypertension) [13]. Individuals who were physically and/or mentally challenged and pregnant women were excluded from the study. The qualitative phase included in-depth interviews with five individuals with controlled hypertension and six with uncontrolled hypertension from the quantitative phase, as well as frontline healthcare providers such as junior health inspectors (JHI), junior public health nurses (JPHN), mid-level service providers (MLSP), accredited social health activists (ASHA), primary health center (PHC) medical officers, and private medical practitioners from both districts, to obtain healthcare provider perspectives.

Sample size and selection process

**Figure 1:**
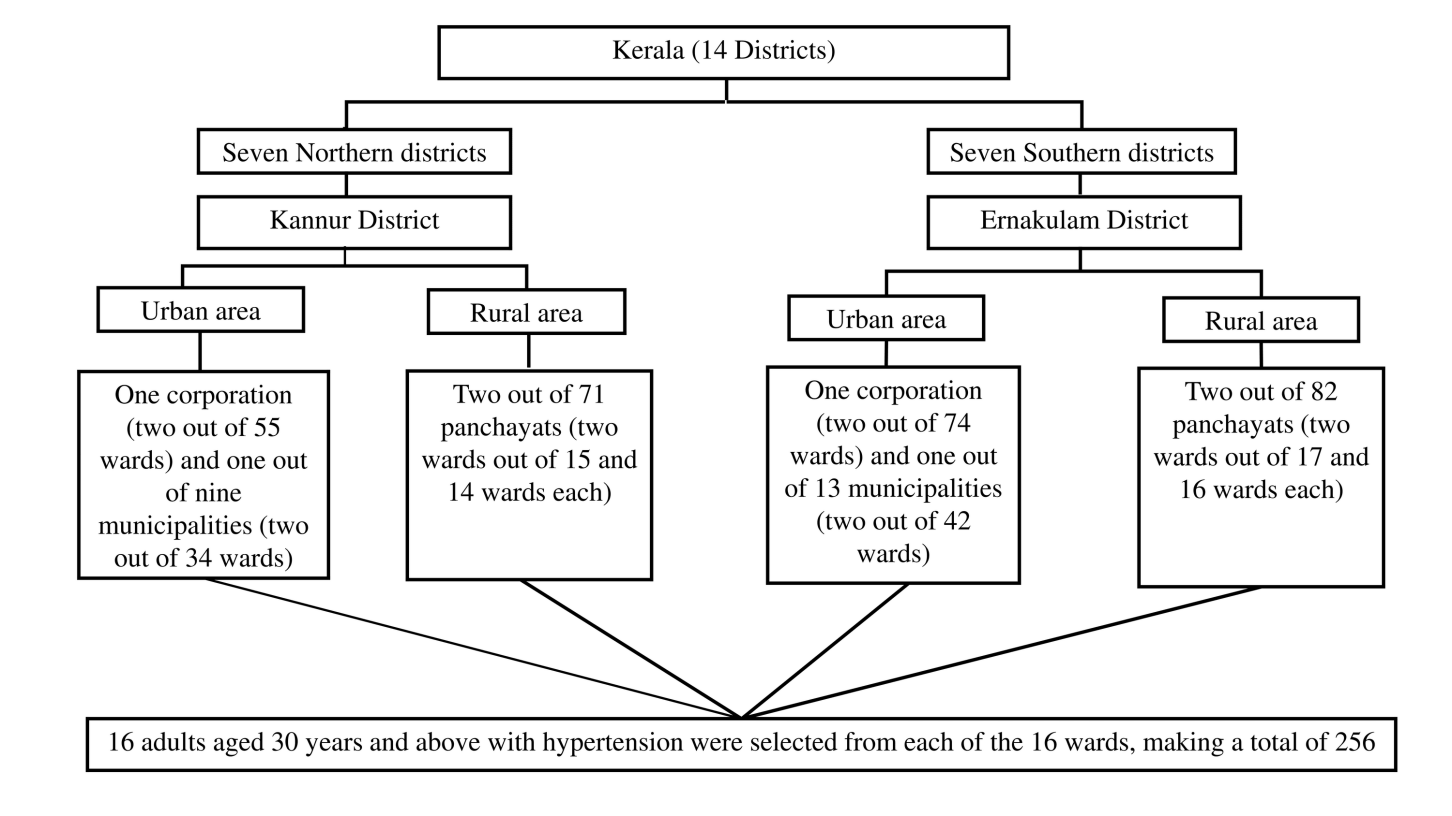
Sample selection process

The quantitative phase required 256 adults with hypertension, based on a 12.4% hypertension control rate [9], 95% confidence level, 5% absolute error, and a design effect of 1.5. A multistage cluster sampling method was used. Stratification was done at the Kerala level by dividing the state into southern and northern districts. Within each district, further stratification was carried out into urban and rural areas, with wards serving as the clusters selected from both. In each cluster, 16 hypertensive adults were selected, with one participant per household chosen using the Kish method (Figure 1).

Of the 525 households approached, 517 individuals (98.5%) agreed to participate and had their blood pressure measured. Given the high and consistent response rates across wards, the likelihood of significant nonresponse bias is minimal. The qualitative phase used purposive sampling for 25 in-depth interviews, ensuring variation until theoretical saturation. Individual interviews with each stakeholder were concluded once no new insights emerged.

Data collection tools and techniques

Data were collected from February 15 to April 15, 2024, by the lead author (PVA). In the quantitative phase, 525 households were approached; 517 (98.5%) consented. Among 517 adults, 256 (49.5%) had hypertension and were interviewed using a pretested World Health Organization (WHO) STEPwise approach to noncommunicable disease (NCD) risk factor surveillance (STEPS) questionnaire [14]. Medication adherence was assessed via the Morisky Green Levine Scale (MGLS) [15]. The survey instrument is attached as an Appendix. The investigator received standardized training from a certified medical professional on proper blood pressure measurement techniques, including appropriate cuff placement, ensuring adequate rest before measurement, and accurate use of the digital device. The OMRON HEM 7120 monitor used in the study was factory-calibrated and routinely checked against a reference device at weekly intervals to ensure consistent accuracy. The average of the last two readings was considered the BP of the individual. Waist circumference was measured with SECA tape.

Study variables

Outcome Variable

Hypertension control: Hypertension control was assessed based on BP measurements taken on the day of the survey. Participants were classified as having controlled hypertension if their SBP was less than 140 mmHg and their DBP was less than 90 mmHg. Those with an SBP of 140 mmHg or higher and/or a DBP of 90 mmHg or higher were considered to have uncontrolled hypertension

Independent Variables

Sociodemographic variables included age, sex, residence (urban/rural), religion, marital status, education, occupation, and socioeconomic status (above poverty line (APL)/below poverty line (BPL)).

Behavioral and clinical variables included tobacco/alcohol use (past month), adequate fruits and/or vegetables consumption (≥5 servings/day), physical activity (<150 minutes moderate/75 minutes vigorous/week), abdominal obesity (≥80 cm women, ≥90 cm men), hypertension medication use, self-reported diabetes/dyslipidemia, and MGLS adherence (low: 3-4; moderate: 1-2; high: 0) [15].

In the qualitative phase, interview guides were developed based on literature [11,12,16,17] and expert consultation (Appendix). Face-to-face interviews (~30 minutes) were conducted in Malayalam, recorded, transcribed, translated to English, and iteratively refined for emerging themes. 

Data analysis

STATA Version 14 was used for statistical analysis. Categorical variables such as sex, residence, religion, marital status, education, occupation, socioeconomic status, current tobacco use, current alcohol use, inadequate fruits and/or vegetables consumption, inadequate physical activity, abdominal obesity, and comorbidities were summarized as frequencies and percentages. Continuous variable for age was summarized as mean (SD). Hypertension status, awareness, treatment, medication adherence, and control of hypertension were expressed as percentages with 95% confidence interval (CI). Age-standardized estimates of hypertension prevalence and control were calculated using the direct method, with the WHO World Standard Population as the reference. The age bands used for standardization were 30-34, 35-39, 40-44, 45-49, 50-54, 55-59, 60-64, 65-69, 70-74, 75-79, 80-84, 85-89, and 90-94 years. For subgroup analyses restricted to individuals aged 30 to 79 years, only the corresponding age groups (30-79) were used. The WHO standard weights for each age group were as follows: 7.61% (30-34), 7.15% (35-39), 6.59% (40-44), 6.04% (45-49), 5.37% (50-54), 4.55% (55-59), 3.72% (60-64), 2.96% (65-69), 2.21% (70-74), 1.52% (75-79), 0.91% (80-84), 0.44% (85-89), and 0.15% (90-94). These standard weights were applied to obtain age-standardized prevalence estimates across study groups. 

Association of hypertension control with the sociodemographic, behavioral, and other characteristics was assessed using binomial regression and crude prevalence ratios (CPRs) with 95% CI. Variables that had a p-value <0.2 in the unadjusted analysis were included in a log binomial regression analysis, and adjusted prevalence ratios (APRs) with 95% CI were calculated. A p-value <0.05 was considered statistically significant.

Qualitative data analysis followed a thematic analysis approach [18]. Audio recordings were transcribed, translated, and inductively coded. Codes were grouped into axial codes and categorized into themes. MS Excel (Microsoft Corporation, Redmond, Washington, United States) was used for listing codes. Triangulation was achieved by collecting data from multiple stakeholder groups to capture diverse perspectives, and by involving two investigators (PVA and SCT) in the analysis to enhance interpretive rigor and reduce individual bias.

Ethical clearance

The study was approved by the Institutional Ethics Committee of Amrita School of Medicine, Kochi (ECASM-AIMS-2024-064), dated January 30, 2024. Written informed consent was obtained from all participants before the data collection. Participation in the study was purely voluntary. All interview transcripts were de-identified by removing names and any potentially identifying information. Confidentiality was maintained through secure data storage, with transcripts stored in password-protected files accessible only to the research team. 

## Results

Quantitative results

Study sample characteristics are given in Table 1.

**Table 1:** Study sample characteristics (n = 256) ^i^Marital status based on the National Family Health Survey (NFHS) classification: widowed, n = 58; separated, n = 1; deserted, n = 1; never married, n = 4; divorced = 0.  ^ii^Homemaker, n = 66; unemployed, n = 17; unable to work, n = 1. ^iii^Based on the classification of socioeconomic status according to the ration cards provided by the government of India.  ^iv^Use of any tobacco product in the last month, ^v^Use of any alcoholic product in the last month, ^vi^>= five servings, ^vii^150 minutes of moderate physical activity or 75 minutes of vigorous physical activity in the last week, ^viii^waist circumference >= 80 cm for women and >= 90 cm for men (based on the World Health Organization (WHO) Asia Pacific guidelines)

Variable	Categories	Males	Females	Total
		(n = 116)	(n = 140)	(n = 256)
		n (%)	n (%)	n (%)
Age in years	>=60	50 (43.1)	75 (53.6)	125 (48.8)
	<60	66 (56.9)	65 (46.4)	131(51.2)
Area of residence	Urban	55 (47.4)	73 (52.1)	128 (50.0)
	Rural	61 (52.6)	67 (47.9)	128 (50.0)
Marital status	Currently married	111 (95.7)	81 (57.9)	192 (75.0)
	Others ^i^	5 (4.3)	59 (42.1)	64 (25.0)
Education	Less than primary schooling	2 (1.7)	14 (10.0)	16 (6.2)
	Primary schooling to higher secondary	87 (75.0)	109 (77.9)	196 (76.6)
	College education and above	27 (23.3)	17 (12.1)	44 (17.2)
Occupation	Currently working	69 (59.5)	20 (14.3)	89 (34.8)
	Pensioner	34 (29.3)	49 (35.0)	83 (32.4)
	Others ^ii^	13 (11.2)	71 (50.7)	84 (32.8)
Socioeconomic status ^iii^	Above poverty line	70 (60.3)	74 (52.9)	144 (56.2)
	Below poverty line	46 (39.7)	66 (47.1)	112 (43.8)
Current tobacco use^ iv^	Yes	49 (42.2)	1 (0.7)	50 (19.5)
	No	67 (57.8)	139 (99.3)	206 (80.5)
Current alcohol use ^v^	Yes	60 (51.7)	1 (0.7)	61(23.8)
	No	56 (48.3)	139 (99.3)	195 (76.2)
Fruits and/or vegetables consumption per day	Adequate ^vi^	15 (12.9)	48 (34.3)	63 (24.6)
	Inadequate	101(87.1)	92 (65.7)	193 (75.4)
Physical activity per week	Adequate ^vii^	18 (15.5)	18 (12.9)	36 (14.1)
	Inadequate	98 (84.5)	122 (87.1)	220 (85.9)
Abdominal obesity	Present ^viii^	90 (77.6)	119 (85.0)	209 (81.6)
	Absent	26 (22.4)	21 (15.0)	47 (18.4)

Over half of our participants were females (n = 140; 54.7%). Age ranged from 30 to 92 years, with a mean of 59.3 (±13.16) years. The hypertension prevalence in our sample population was 49.51% (95% CI = 45.23-53.81) (Table 2).

**Table 2:** Prevalence, awareness, treatment, medication adherence and control of hypertension CI: confidence interval ^i^Based on the Morisky Green Levine's Medication Adherence Four-Item Scale (MGLS); scores ranging from 0 (high adherence) to 4 (low adherence)

Variable	n (%)	95% CI
Hypertension prevalence (N = 517)	256 (49.51)	45.23-53.81
Hypertension awareness (N = 256)	181 (70.70)	64.86-75.94
Currently on medication for hypertension (N = 256)	145 (56.64)	50.52-62.57
High anti-hypertensive medication adherence^i ^ (N = 145)	45 (31.03)	24.08-38.97
Controlled hypertension among all hypertensives (N = 256)	59 (23.04)	18.31-28.58
Controlled hypertension among those who were aware of their hypertension (N = 181)	59 (32.59)	26.19-39.72
Controlled hypertension among treated hypertensives (N = 145)	59 (40.68)	33.03-48.83

The age-standardized prevalence based on the WHO world standard population was 48.17%. The age-standardized hypertension prevalence and control among adults aged 30-79 years, the age group suggested by the WHO, were 47.35% (n = 256) and 15.47% (n = 59), respectively. Distribution of participants according to hypertension status is provided in Table 3.

**Table 3:** Distribution of participants according to hypertension status (N = 256) ^i^Systolic blood pressure (SBP) <140 mmHg and diastolic blood pressure (DBP) <90 mmHg. ^ii^SBP 140-159 mmHg and/or DBP 90-99 mmHg. ^iii^SBP >=160 mmHg and/or DBP >=100 mmHg

Hypertension status	n (%)
Controlled^i^	59 (23.04)
Stage 1 hypertension^ii^	150 (58.60)
Stage 2 hypertension^iii^	47 (18.36)

Older age of 60 years and above (APR = 2.91; 95% CI = 1.84-4.59), females (APR = 1.97; 95% CI = 1.26-3.10), adequate fruits and vegetables consumption (APR = 6.51; 95% CI = 3.75-11.30), adequate physical activity (APR = 2.22; 95% CI = 1.44-3.44), absence of abdominal obesity (APR = 1.87; 95% CI = 1.22-2.88) and absence of comorbidities (APR = 1.83; 95% CI = 1.22-2.73) were significantly associated with hypertension control (Table 4).

**Table 4:** Factors associated with hypertension control: results of multivariable analysis (log binomial regression) (n = 256) UPR: unadjusted prevalence ratio; APR: adjusted prevalence ratio; CI: confidence interval ^i^Diabetes, dyslipidemia. Bold values indicate statistically significant at p < 0.05. Variables that had a p-value <0.2 in the unadjusted analysis were included in the adjusted analysis

Variable	n	Controlled	UPR (95%CI)	APR (95% CI)	p-value
		n (%)			
Age in years					
>=60	125	42 (33.6)	2.58 (1.55-4.30)	2.91 (1.84-4.59)	<0.001
<60	131	17 (13.0)	Reference	Reference	-
Sex					
Female	140	40 (28.6)	1.74 (1.07-2.84)	1.97 (1.26-3.10)	0.003
Male	116	19 (16.4)	Reference	Reference	-
Marital status					
Other marital status	64	20 (31.3)	1.53 (0.97-2.43)	1.04 (0.61-1.77)	0.877
Currently married	192	39 (20.3)	Reference	Reference	-
Occupation					
Pensioner	83	28 (33.7)	2.30 (1.28-4.14)	1.13 (0.66-1.91)	0.646
Other occupation	84	18 (21.4)	1.46 (0.76-2.80)	0.83 (0.49- 1.39)	0.484
Currently working	89	13 (14.6)	Reference	Reference	-
Socioeconomic status					
Above poverty line	144	40 (27.8)	1.63 (1.01-2.66)	1.03 (0.64-1.66)	0.889
Below poverty line	112	19 (17.0)	Reference	Reference	-
Current tobacco use					
No	206	54 (26.2)	2.62 (1.10-6.21)	1.19(0.66-2.13)	0.557
Yes	50	5 (10)	Reference	Reference	-
Current alcohol use					
No	195	53 (27.2)	2.76 (1.24-6.11)	1.11 (0.59-2.08)	0.742
Yes	61	6 (9.8)	Reference	Reference	-
Fruits and/or vegetables consumption per day					
Adequate	63	46 (73.0)	10.84 (6.27-18.71)	6.51 (3.75-11.30)	<0.001
Inadequate	193	13 (6.7)	Reference	Reference	-
Physical activity per week					
Adequate	36	28 (77.8)	5.51 (3.81-7.99)	2.22 (1.44-3.44)	<0.001
Inadequate	220	31 (14.1)	Reference	Reference	-
Abdominal obesity					
Absent	47	29 (61.7)	4.29 (2.87-6.41)	1.87 (1.22-2.88)	0.004
Present	209	30 (14.4)	Reference	Reference	-
Comorbidities ^i^					
Absent	133	36 (27.1)	1.44 (0.91-2.29)	1.83 (1.22-2.73)	0.003
Present	123	23 (18.7)	Reference	Reference	-

Qualitative results

**Figure 2:**
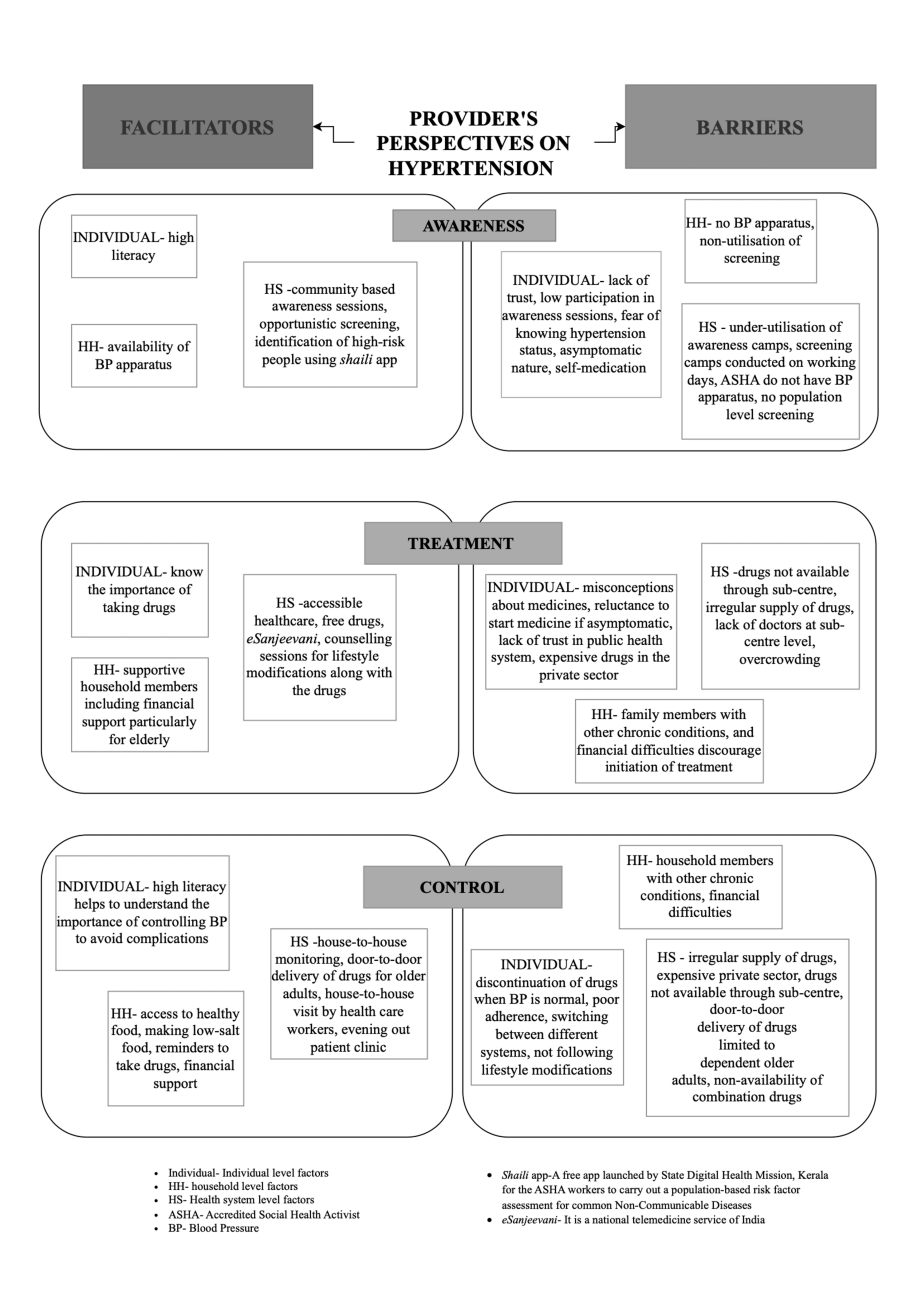
Facilitators and barriers of hypertension control: provider’s perspective

Findings highlight perspectives on hypertension control from individuals and healthcare providers. Key themes were barriers and facilitators to control, categorized into awareness, treatment, and control at individual, household, and health system levels (Table 5, Figure 2).

**Table 5:** Key themes identified with definitions and illustrative quotes PHC: primary health center; BP: blood pressure; JPHN: junior public health nurse; NCD: noncommunicable disease; CHWs: community health workers; MLSP: mid-level service provider; JHI: junior health inspector; ASHA: accredited social health activist; CBPs: community-based programs P1: controlled hypertensive, male, 59 years; P2: controlled hypertensive, female, 50 years; P3: uncontrolled hypertensive, female, 62 years; P4: uncontrolled hypertensive, male, 71 years; P5: controlled hypertensive, female, 68 years; P6: controlled hypertensive, male, 72 years; P7: uncontrolled hypertensive, male, 52 years; P8: uncontrolled hypertensive, female, 65 years; P9: controlled hypertensive, female, 80 years; P10: uncontrolled hypertensive, male, 45 years

Facilitators of hypertension control under three subthemes			Theme	Barriers to hypertension control under three subthemes		
Individual level (quotes)	Household level (quotes)	Health system level (quotes)	(Description/meaning)	Individual level (quotes)	Household level (quotes)	Health system level (quotes)
“Individuals with sufficient knowledge regarding hypertension are naturally inclined to adhere to regular screening, which aids in the early detection and prevention of conditions.”-PHC Medical Officer 1. “I don’t fear the side effects of medicines. We have doctors to guide us, and they know everything, so we can easily approach them to clarify any doubts. So, I never pull myself back from checking my BP, and that helped me identify hypertension at an early stage. I’m continuing my treatment without any hesitation.”-P1	"In some houses, especially where there are elderly or diabetic patients, they already have a BP apparatus. If someone in the family knows how to use it, they check BP regularly at home, and that's how they come to know early if something is wrong."-JPHN 1. “My husband checks my BP regularly. One day, he found it very high, so we consulted a doctor immediately. Since then, I’ve been on medication for hypertension.”-P2	"Weekly ward-wise NCD clinics, Arogya Mela, Vishayadhishttitha clinic, and field visits by CHWs enable screening, monitoring, and awareness. This has led to early hypertension detection, fewer complications, and noticeable improvements in the community and family health center visits over the past year."-PHC Medical Officer 1. "The MLSP post is essential and has strong potential to impact community health, especially in NCD control positively."-PHC Medical Officer 2	Awareness (knowledge regarding the hypertension status of individuals) refers to an individual's awareness of whether they have high blood pressure, their understanding of what hypertension means, its potential health risks, and the importance of regular monitoring for early detection and management	“Working people often complain that they are unable to attend community-based screening and awareness programmes, like the NCD clinic, because they are held on Thursdays, which is a working day. That is why we are getting fewer new cases.”-JHI 1 “Before the attack, I didn’t have any problems/ symptoms, and I was able to do all the work like cooking food at home, feeding the cow, milking, and carrying milk to many houses. It was like non-stop work. So, I never checked my BP and was not aware that my blood pressure was high. That is why I didn’t take any treatment before.”-P3	“In some houses, they even have a BP apparatus, but there’s no one to help them use it. Especially for elderly people-they want to check their BP, but they can’t do it on their own.”-ASHA 1 “There is a BP apparatus at home, but I don’t have anyone to check my BP for me, so I’m not using it.”-P4	“It is better to conduct health education for the adolescent group at school or elsewhere, as children can influence their parents too. But there are not enough human resources for executing such programmes.”-PHC Medical Officer 1
“There are patients who understand the seriousness of high BP. They know that starting treatment early can prevent future complications, so they don’t delay-they come, ask for advice, and begin medication without resistance.”-Private Medical Practitioner 4. “I walk four kilometres daily (morning + evening). That is what matters most in my BP control.”-P5	“For people in the priority group, we visit their homes to check blood pressure. If it’s high, we record the reading and any symptoms in their NCD book. Then a family member can take the book to the health center, consult the doctor, and collect the prescribed medicines.”-MLSP 1. “My wife always takes care of me by managing my diet, doctor’s appointments, buying medicines, and accompanying me to the hospital.”-P6	“ASHAs follow up the referred cases from the NCD clinic who do not come here (PHC).”-PHC Medical officer 2. “Now, the evening OP, which is going on in the PHC, enhances the convenience of working groups to utilize the public health facilities.”-PHC Medical Officer 1	Treatment (Initiation of antihypertensive medication along with lifestyle modification) refers to the initiation of antihypertensive medication and lifestyle changes like diet, exercise, and stress control. It involves factors such as trust in doctors, family support, and personal beliefs, highlighting the need for accessible and ongoing care.	"Patients often resist treatment due to concerns about lifelong medication, preferring lifestyle changes. But with high BP like 180 mmHg, both medication and lifestyle advice are necessary."-PHC Medical Officer 2. “The homeopathy doctor himself has told me to take allopathy medicine for some time and let the BP be under control. But I have not started taking allopathic medicine yet because I’m not interested in taking medicine for the rest of my life. I think if I am taking homeopathic medicine, it is possible to stop once BP becomes normal.”-P7	“There are many parents who have children outside, like husband and wife only. In some cases, the husband has many diseases, then the wife takes care of the husband, while the wife does not pay much for her hypertension, so it is left unattended.”-Private Medical Practitioner 1. “I live alone, and there’s no one to take care of my diet or treatment. I’m not able to buy fruits and vegetables, and I can’t cook either. So, I usually prefer rice porridge, pickle, and papad.”-P8	"I think the absence of an on-site doctor with no medicine supply is one of the main reasons for the lower participation of people in CBPs like NCD camp/clinic."-MLSP 2 "People often avoid buying medicines as the PHC is far and overcrowded. If pharmacists are posted at the Janakeeya Arogya Kendra, this issue can be avoided."-MLSP 2
“Spending 10 to 15 minutes with each patient helps build trust, which improves communication, treatment adherence, and encourages them to follow lifestyle changes. This personal approach leads to better compliance.”-Private Medical Practitioner 3. “I visit my doctor every month and buy medicines after doing the necessary tests to check if any dose adjustment is needed. Based on the readings, the doctor makes changes, and the treatment helps me maintain normal BP. I have strong faith in the doctor and feel reassured during visits. The first thing he asks is whether I’m exercising.”-P5	"Many elderly forget their medication, so I recommend using a labeled pill box supervised by family or caregivers to help them keep track effectively."-Private Medical Practitioner 3. “My daughter takes me to the doctor on time and bought me a pill box with medicines for morning, noon, and night. I keep it on the dining table, so I remember to take it before and after meals. The children explained everything clearly and helped me initially, so I never faced any difficulty using it.”-P9	"In PHC, we need to prioritize commonly available drugs like Amlodipine, Telmisartan, Enalapril, and HCT for initial treatment to ensure continuous, affordable care and avoid therapy disruptions."-PHC Medical Officer 1	Control (Adherence to the medical and lifestyle recommendations for hypertension) refers to how consistently individuals follow medication and lifestyle recommendations to manage BP. It is influenced by support systems, affordability, access to care, and personal habits	“When asked to eat fruits, some people say that they've diabetes, so they can't eat them. But the reality is that only certain fruits should not be eaten by diabetics. Apple, guava, etc, can be eaten.”-Private Medical Practitioner 2. "Many stop medication once BP normalizes and return only with symptoms. We advise regular monitoring, not stopping without consultation, and reducing dosage gradually if BP is controlled."-PHC Medical Officer 2. “I know that papad, pickle, dried fish, etc, causes hypertension. But I eat all of them, none of them, daily. Fish curry and fried fish are mostly eaten, especially dried fish.”-P10	"In my area, it’s mostly elderly parents living alone while their children are away. They often do not take their food and medicine on time. They told me that if their children were around, they would remind them."-ASHA 2 “To get medicine for free, I have to reach the medical college at 5 am and collect the token. No one here in my house gets up and leaves at that time.”-P3	"We need more staff. During monsoon, each doctor sees 150–200 patients a day, allowing little time for follow-ups. Quick consultations affect service quality. With more doctors, we could also attend NCD clinics or camps in shifts."-PHC Medical Officer 1. "Since combination drugs aren't provided at the PHC, patients get multiple tablets instead of one. Some end up taking only one, leading to improper dosing."-MLSP 1

Perspectives of individuals with hypertension

Free drug supply from the public health system, ASHA’s household visits, and home drug delivery for older adults were facilitators. Barriers included access issues due to distance and waiting times, provider attitudes, lack of awareness about regular medication and lifestyle changes, irregular drug supply, drug provided only for one month, high private-sector costs, pill burden, neglect due to symptom absence, and inadequate family support.

Providers’ perspectives

Facilitators for Awareness, Treatment, and Control of Hypertension

At the individual level, high literacy acted as a facilitator in having awareness as well as control of hypertension, while awareness of the complications of hypertension was identified as a treatment facilitator. At the household level, a facilitator for awareness was having a BP apparatus, which helped in monitoring even nonhypertensive adults in the household and helped early detection of hypertension. At the same time, supportive family members acted as a major facilitator for treatment initiation, the presence of household support for home BP monitoring, timely medication refills with reminders, adherence to a salt-restricted diet, accessing vegetables and fruits, and financing treatment acted as facilitators for control.

At the health system level, awareness was enhanced by NCD clinics, opportunistic BP screening, and the Shaili app, which identifies high-risk individuals and directs them to PHCs. Free medicines, consultations, and e-health services, such as eSanjeevani, with MLSP support facilitated treatment. Control was supported through BP monitoring of dependent older adults during MLSP visits, door-to-door medicine delivery, ASHA-led counseling, and evening outpatient services at FHCs.

“Home BP monitoring is highly recommended nowadays. In households with a BP monitor, all adult members are likely to check their BP, enabling early detection of hypertension.”-Private Medical Practitioner 3 

"The Shaili App, managed by ASHAs, assesses NCD risk for individuals aged 30 years and above. Those at risk are identified through a score and notified to visit the PHC to check their BP.”-PHC Medical Officer 1

Barriers to Awareness, Treatment, and Control of Hypertension

Individual-level barriers to awareness included mistrust in public healthcare, especially among younger and working individuals, leading to poor attendance at awareness sessions and underutilized screening camps. Apart from these, fear of BP diagnosis, self-medication, and asymptomatic hypertension are common. Treatment barriers involved misconceptions about side effects, lifelong medication, drug-induced complications, reluctance due to the absence of symptoms, and distrust in public healthcare. Control barriers included switching treatments without BP monitoring, stopping medication when BP normalized, delayed follow-ups, pill burden, rushed consultations, and unreviewed medication use. Misconceptions about low-intensity chores as exercise, high fruit costs, and working women’s work-life balance struggles contributed to inadequate diets and physical activity.

At the household level, not having a BP apparatus was noted as a barrier to awareness. Household financial problems and the presence of household members with serious NCDs, such as cancer, stroke, or kidney disease, along with household financial hardship, often discouraged asymptomatic hypertensive patients from initiating treatment or neglecting their medication. For control, lack of support from family members deterred hypertensives’ adherence to treatment and lifestyle modifications.

At the health system level, awareness barriers included underutilized screening camps, insufficient funds, inadequate logistics for field activities, and a lack of BP apparatus with ASHAs, reducing screening effectiveness. Treatment initiation was hindered by irregular medicine supply, doctor shortages, insufficient medicines at peripheral facilities and NCD camps, overcrowding, and distant PHCs with poor doctor-patient ratios, causing long wait times and poor service quality. Network issues also disrupted eSanjeevani consultations.

"I often see people switching between allopathic, homeopathic, and ayurvedic medicines, with some believing that herbal remedies offer immediate, lasting relief and can replace lifelong allopathic treatment, which can result in uncontrolled hypertension."-ASHA 2

"People are more worried about the lifelong treatment, medication side effects, dependency, rather than the complications of hypertension itself. Efforts to address these problems of the hypertensive patients are required."-Private Medical Practitioner 2

## Discussion

In our study of 256 hypertensive adults aged ≥30 years, the control rate was 23.04%. Despite an improvement from previous rates (8.8% [17] in 2003, 11.4% [19] in 2000, 12.4% [9] in 2019, and 18.5% [20] in 2015), hypertension control in Kerala remains low. Our findings align with other Kerala studies reporting control rates of 23.5% [10] and 24% [11] among adults aged 40-70 and ≥60 years, respectively.

Older adults in our study had better hypertension control than younger adults, consistent with other studies [6,8]. This may be attributed to better compliance with treatment [21]. However, some studies reported a lower likelihood of control in older adults [22]. Qualitative study revealed that older adults followed medical and lifestyle recommendations more diligently than younger adults, and those with other chronic diseases received medications at home. Younger adults showed reluctance toward medication adherence and participation in community-based hypertension programs due to mistrust in the public health system and work commitments. Integrating routine health assessments into workplaces, organizing community-based programs on holidays, and increasing awareness sessions in colleges may improve hypertension control among younger adults.

Females exhibited better control rates than males, likely due to greater awareness from antenatal services [1], more frequent screening [1], and lower tobacco/alcohol use, as observed in our study and another study conducted in a South Asian country [23]. These findings align with some studies [10,20] but contrast with research in the United Kingdom (UK) [24], which reported a lower hypertension control rate in females compared to males. Qualitative findings indicated greater health-seeking behavior and participation in community health programs among females.

Adequate vegetable and fruit consumption correlated significantly with hypertension control, consistent with a systematic review [25] and a Kerala study [26]. Nutrients in fruits and vegetables, like fiber, potassium, and vitamins, may help lower BP, alongside maintaining a healthy weight [27]. However, financial constraints hinder consumption, necessitating nutrition education, subsidized access, and promotion of locally available options. Other studies reported no significant association [11]. Qualitative research highlighted reluctance to eat fruits and vegetables due to financial constraints in purchasing them, which is consistent with another study [28]. Nutrition education, better access, and availability of fruits and vegetables at a subsidized rate, and encouraging consumption of locally available and cheaper fruits and vegetables are likely to increase fruit and vegetable consumption.

Adequate physical activity was significantly associated with hypertension control, as seen in several studies [11,29]. Exercise reduces weight, enhances renal function, and strengthens the heart [29]. Conversely, a study conducted in the UK [24] found conflicting results. Promoting physical activity at home, workplaces, and public spaces may enhance adherence.

Absence of abdominal obesity was linked to better hypertension control, aligning with studies [5,29]. Abdominal obesity, linked to poor diet and physical inactivity, increases cardiac output and arteriolar resistance, which hinders hypertension management [29]. However, one study reported no significant association [30]. Qualitative research found that working women struggle with work-life balance, leading to fast-food reliance, reduced physical activity, and obesity.

Participants without comorbidities showed better control rates, consistent with previous studies [23,26,30]. However, a study [31] argues that comorbidities facilitate frequent monitoring, improving control. Diabetes, dyslipidemia, and kidney disease complicate hypertension management [29]. Qualitative analysis highlighted the pill burden as a barrier to adherence.

High adherence to antihypertensive medication positively correlated with hypertension control, supported by prior studies [26,29]. Qualitative findings revealed awareness, household support, and structured treatment as facilitators, while lack of awareness and inadequate healthcare support were barriers [11]. Facilitators include awareness of complications and control measures of hypertension and support from household members in facilitating treatment. Barriers include a lack of awareness of complications of uncontrolled hypertension, a lack of support from household members, and the healthcare system.

One of the strengths of our study was that we used a mixed-methods study that enabled us to estimate the current hypertension control rate from a representative sample of the 33.4 million Kerala population and collected information on both hypertensives and providers’ perspectives using in-depth interviews. Although hypertensives’ perspectives were reported early from Kerala, most of which are also reported in this study, providers’ perspectives on hypertension control were not reported from Kerala or India. Limitations include the cross-sectional design of the survey, from which causality cannot be established, and self-reported information on diet and physical activity, and a few other variables.

The findings of this study hold significant policy relevance for Kerala’s health system, particularly within the framework of the National Program for Noncommunicable Diseases (NP-NCD). Despite Kerala’s high burden of hypertension and relatively advanced health infrastructure, the persistently low control rates underscore the need to move beyond screening and treatment initiation toward sustained, person-centered care. The barriers identified, such as limited treatment adherence, fragmented follow-up, and distrust in public services, highlight the importance of reinforcing community-based support through ASHAs, JPHNs, and MLSPs. These cadres can play a crucial role in counselling, regular follow-up, and linking patients to continuous care. Kerala’s decentralized health governance and strong primary care network offer an ideal platform to pilot district-level interventions tailored to local contexts. In comparison, Tamil Nadu and Gujarat have implemented population-based NCD registries and follow-up mechanisms with some success. Internationally, countries like Sri Lanka and Thailand have improved hypertension control through integrated primary care and task-shifting strategies. By embedding similar approaches within NP-NCD, Kerala can optimize its existing system to address hypertension more effectively and serve as a scalable model for other Indian states and LMICs facing similar epidemiological transitions. Future research should include longitudinal studies to track BP control and adherence patterns over time, providing insights into long-term hypertension management. Intervention trials targeting identified gaps, such as poor follow-up, low adherence, and distrust in public services, are also essential. These could test community-based support models, digital reminders, or task-sharing with frontline workers. Piloting such strategies within NP-NCD settings can generate evidence for scalable solutions. Additionally, further studies should explore provider-level challenges and system-level barriers to sustained hypertension control.

## Conclusions

Despite Kerala’s favorable health indicators, hypertension control remains low at 23%. Better control was observed among older adults, females, individuals with adequate diet and physical activity, those without abdominal obesity, and those free from comorbidities, findings that align with previous research from the region. Providers identified key barriers such as irregular drug supply, limited outreach to younger and working populations, and the financial burden of treatment in the private sector.

These findings suggest that targeted strategies, such as improving drug supply continuity, enhancing community outreach, and reducing cost-related barriers, may support better hypertension control. Situating these insights within established frameworks like the Chronic Care Model and the WHO Health Systems Responsiveness framework helps contextualize the interplay of individual, provider, and system-level influences. This theoretical grounding strengthens the relevance of our observations and can guide future interventions and policy efforts aimed at improving chronic disease management in similar settings.

## Appendices

Survey instrument

Sociodemographic Factors

1. Name and address of the participant (including phone number if they are willing):

2. Age of the participant (in completed years):

3. Gender of the participant:

(a) Male

(b) Female

(c) Transgender

4. Place of residence:

(a) Urban

(b) Rural

5. Marital status:

(a) Currently married

(b) Widowed

(c) Divorced

(d) Separated

(e) Deserted

(f) Never married

6. Highest level of education:

(a) Less than primary schooling

(b) Primary schooling to higher secondary

(c) College education and above

7. Occupational status:

(a) Currently working

(b) Homemaker

(c) Unable to work

(d) Pensioner

(e) Unemployed

8. Socioeconomic status:

(a) APL

(b) BPL

Behavioral Risk Factors

1. Did you use any tobacco products in the last one month?

(a) Yes

(b) No                      

2. Did you use any alcoholic products in the last one month?

(a) Yes

(b) No

3. In a typical week, how many days do you consume any fruits? ______________

(Number of days)

4. How many servings of fruits do you consume on those days? _________________________

(Number of servings)

5. In a typical week, how many days do you consume any vegetables? ______________

(Number of days)

6. How many servings of vegetables do you consume on those days? _________________

(Number of servings)

7. Did you do 150 minutes of moderate physical activity or 75 minutes of vigorous physical

activity in the last one week?

(a) Yes

(b) No

8. Has any healthcare provider told you that you have high blood pressure?

(a) Yes

(b) No

9. Are you currently taking any medication for hypertension?

(a) Yes

(b) No

10. Has any healthcare provider told you that you have diabetes?

(a) Yes

(b) No

11. Has any healthcare provider told you that you have dyslipidaemia?

(a) Yes

(b) No

Anthropometric Measurement

1. Waist circumference of the participant (in centimeters):

Clinical measures

1. Systolic BP (in mmHg) of the participant:

Reading 1: ___________mm of Hg

Reading 2: ___________mm of Hg

Reading 3: ___________mm of Hg

2. Diastolic BP (in mmHg) of the participant:

Reading 1: ___________mm of Hg

Reading 2: ___________mm of Hg

Reading 3: ___________mm of Hg

3. Pulse rate of the participant:

Reading 1: __________rate/ minute

Reading 2: __________rate/ minute

Reading 3: __________rate/ minute

Morisky Green Levine Medication Adherence Scale (4 Items)

1) Do you ever forget to take your anti-hypertensive medicine?

(a) Yes

(b) No

2) Are you careless at times about taking your anti-hypertensive medicine?

(a)Yes

(b) No

3) When you feel better, do you sometimes stop taking your medicine?

(a) Yes

(b) No

4) Sometimes if you feel worse when you take the medicine, do you stop taking it?

(a) Yes

(b) No

In-depth interview guide

In-Depth Interview Guide for Individuals With Hypertension

1.     Can you please explain how you realised you have hypertension?

(Probe: experience, diagnosis, source of care-medical providers, traditional healers/other nonmedical providers, reason for your hypertension in your opinion)

2.     What is your hypertension level now? (whether on medicines, follow-up)

If it is controlled, can you please elaborate different strategies that you follow to manage your high

blood pressure? (Probe: motivation for drug adherence (reminder like alarm/partner/family member/friend/mobile app), routine BP monitoring (clinic/home), treatment follow-up regularity (family and health care system support), lifestyle modifications)

3.     What, according to you, has mostly helped in managing your condition effectively? (probe: personal motivation, family support, health system factors)

4.     Do you think you faced any challenges in managing the condition? Can you explain?

5.     How do you think you can overcome these challenges or what additional support do you need to manage your condition?

If it is uncontrolled, why do you think you are unable to control your condition? 

(Probe: (individual) knowledge, attitude and belief about hypertension and treatment-need for lifelong treatment, alternative systems, claim of complete cure, no symptoms etc.; drug intake and follow-up regularity; lack of trust in healthcare system; work commitment; dietary and other lifestyle factors abstinence), social (family support-emotional/monetary/physical), organizational (accessibility, affordability and availability of treatment and medications), community factors (wrong guidance by the society))

6.     Can you suggest some measures which can help you achieve better control of your condition/ high BP?

7.     Do you want to share anything which we missed discussing?

In-Depth Interview Guide for Healthcare Personnel

Questions for PHC Medical Officers and Private Medical Practitioners

1.     Do you think there is a high prevalence of hypertension in this area? If yes, why do you think there is a high prevalence? (Probe: control rate, patient-doctor relationship; medication adherence, follow-up, diet, exercise, other lifestyle factors; conflicts with patients; lack of support)

2.     According to you, what are your suggestions to tackle this situation of high prevalence of hypertension? (Probe for each of the reasons he/she has suggested for as reasons for high prevalence)

3.     Is there any community-based programmes or resources available to adults with hypertension and their caregivers/ family members? (Probe: If yes, explain; If no, why not)

4.     Is there a need to change the current guidelines for hypertension diagnosis and management you are following? [probe: If yes, explain]

5.     What are the measures we can adopt to actively involve the community in the hypertension prevention and control? 

6.     What are your thoughts about the sustainability of hypertension management in the community? What would make this approach more sustainable? 

Questions for MLSPs, JHIs, JPHNs, and ASHAs

1.     Are there any community-based programmes led by (MLSPs/JHIs/JPHNs/ASHAs) for the control of hypertension? If yes, what are all the activities under the programme? (Probe: meeting, house visit, BP monitoring, education and motivation, medicine supply, etc.)

2.     What are the challenges faced in implementing the activities under the programme? (Probe: involvement of community, resource availability, incentives, supervision and assistance, training sessions, etc.)

3.     From your experience, what could be done to make the lives of people suffering from hypertension easier? (Probe: suggestions (in prevention, diagnosis, and treatment))

## References

[1] NCD Risk Factor Collaboration (NCD-RisC) (2021). Worldwide trends in hypertension prevalence and progress in treatment and control from 1990 to 2019: a pooled analysis of 1201 population-representative studies with 104 million participants. Lancet.

[2] Varghese JS, Venkateshmurthy NS, Sudharsanan N (2023). Hypertension diagnosis, treatment, and control in India. JAMA Netw Open.

[3] Mills KT, Bundy JD, Kelly TN (2016). Global disparities of hypertension prevalence and control: a systematic analysis of population-based studies from 90 countries. Circulation.

[4] (2024). Global burden of disease (GBD). https://www.healthdata.org/research-analysis/gbd.

[5] Anjana RM, Unnikrishnan R, Deepa M (2023). Metabolic non-communicable disease health report of India: the ICMR-INDIAB national cross-sectional study (ICMR-INDIAB-17). Lancet Diabetes Endocrinol.

[6] Amarchand R, Kulothungan V, Krishnan A, Mathur P (2023). Hypertension treatment cascade in India: results from National Noncommunicable Disease Monitoring Survey. J Hum Hypertens.

[7] Kearney PM, Whelton M, Reynolds K (2005). Global burden of hypertension: analysis of worldwide data. Lancet.

[8] Gupta R, Gaur K, Ahuja S, Anjana RM (2024). Recent studies on hypertension prevalence and control in India 2023. Hypertens Res.

[9] Sarma PS, Sadanandan R, Thulaseedharan JV (2019). Prevalence of risk factors of non-communicable diseases in Kerala, India: results of a cross-sectional study. BMJ Open.

[10] Geevar Z, Krishnan MN, Venugopal K (2022). Prevalence, awareness, treatment, and control of hypertension in young adults (20-39 years) in Kerala, South India. Front Cardiovasc Med.

[11] Maniyara K, Kodali PB, Thankappan KR (2023). Prevalence, awareness, treatment, control and correlates of prevalence and control of hypertension among older adults in Kerala: a mixed methods study. Indian Heart J.

[12] Ravindranath R, Sarma PS, Sivasankaran S, Thankappan KR, Jeemon P (2024). Voices of care: unveiling patient journeys in primary care for hypertension and diabetes management in Kerala, India. Front Public Health.

[13] Chobanian AV, Bakris GL, Black HR (2003). Seventh report of the Joint National Committee on Prevention, Detection, Evaluation, and Treatment of High Blood Pressure. Hypertension.

[14] (2024). Noncommunicable disease surveillance, monitoring and reporting. https://www.who.int/teams/noncommunicable-diseases/surveillance/systems-tools/steps/instrument.

[15] Beyhaghi H, Reeve BB, Rodgers JE, Stearns SC (2016). Psychometric Properties of the Four-Item Morisky Green Levine Medication Adherence Scale among atherosclerosis risk in communities (ARIC) study participants. Value Health.

[16] Riddell MA, Mini GK, Joshi R (2021). ASHA-led community-based groups to support control of hypertension in rural India are feasible and potentially scalable. Front Med (Lausanne).

[17] Zachariah MG, Thankappan KR, Alex SC, Sarma PS, Vasan RS (2003). Prevalence, correlates, awareness, treatment, and control of hypertension in a middle-aged urban population in Kerala. Indian Heart J.

[18] Nowell LS, Jaffee KD, White DE (2017). Thematic analysis: striving to meet the trustworthiness criteria. Int J Qual Methods.

[19] Kalavathy MC, Thankappan KR, Sarma PS, Vasan RS (2000). Prevalence, awareness, treatment and control of hypertension in an elderly community-based sample in Kerala, India. Natl Med J India.

[20] Sebastian NM, Jesha MM, Haveri SP (2016). Hypertension in Kerala: a study of prevalence, control, and knowledge among adults. Int J Med Sci Public Health.

[21] Cao Y, Sathish T, Haregu T, Wen Y, de Mello GT, Kapoor N, Oldenburg B (2021). Factors associated with hypertension awareness, treatment, and control among adults in Kerala, India. Front Public Health.

[22] Mutneja E, Yadav R, Dey AB, Gupta P (2020). Frequency and predictors of compliance among patients taking antihypertensive medicines. Indian Heart J.

[23] Prenissl J, Manne-Goehler J, Jaacks LM (2019). Hypertension screening, awareness, treatment, and control in India: a nationally representative cross-sectional study among individuals aged 15 to 49 years. PLoS Med.

[24] Tapela N, Collister J, Clifton L, Turnbull I, Rahimi K, Hunter DJ (2021). Prevalence and determinants of hypertension control among almost 100 000 treated adults in the UK. Open Heart.

[25] Madsen H, Sen A, Aune D (2023). Fruit and vegetable consumption and the risk of hypertension: a systematic review and meta-analysis of prospective studies. Eur J Nutr.

[26] Lalu JS, John A, Paul N (2021). Prevalence and risk factors of uncontrolled hypertension in the urban population of Kerala. Int J Community Med Public Health.

[27] Wang L, Manson JE, Gaziano JM, Buring JE, Sesso HD (2012). Fruit and vegetable intake and the risk of hypertension in middle-aged and older women. Am J Hypertens.

[28] Yusuf S, Joseph P, Rangarajan S (2020). Modifiable risk factors, cardiovascular disease, and mortality in 155722 individuals from 21 high-income, middle-income, and low-income countries (PURE): a prospective cohort study. Lancet.

[29] Fekadu G, Adamu A, Gebre M (2020). Magnitude and determinants of uncontrolled blood pressure among adult hypertensive patients on follow-up at Nekemte Referral Hospital, Western Ethiopia. Integr Blood Press Control.

[30] Paul A, Kini SB, Kumar A, Mallya SD (2023). Determinants of blood pressure control among hypertensive patients of rural areas in a South Indian State: a community based cross sectional study. Malays Fam Physician.

[31] Mohanty SK, Pedgaonkar SP, Upadhyay AK (2021). Awareness, treatment, and control of hypertension in adults aged 45 years and over and their spouses in India: a nationally representative cross-sectional study. PLoS Med.

